# Effects of Fluorine on Neutrophil Extracellular Trap Formation through Regulating AMPK/p38 Signaling Pathway

**DOI:** 10.1155/2021/6693921

**Published:** 2021-08-02

**Authors:** Yanyan Song, Yue Zhang, Peijun Zhang, Peng Yu, Xinchi Shang, Yuting Lu, Yuehong Li, Hang Gao

**Affiliations:** ^1^Department of Nephrology, The Second Hospital of Jilin University, Changchun 130021, China; ^2^College of Animal Science and Technology, Jilin Agricultural University, Changchun 130118, China; ^3^Department of Bone and Joint Surgery, The First Hospital of Jilin University, Changchun 130021, China

## Abstract

Fluorine is an important trace element that is widely dispersed, and studies showed that fluorine could cause severe toxicity to fish. The aim of this study was to investigate the effects of fluorine on neutrophil extracellular trap (NET) formation in common carp and clarify the possible mechanism. The neutrophils were isolated and exposed to 0.25, 0.5, or 1 mM sodium fluoride (NaF). The results showed that NaF could induce the formation of NETs which exhibited a DNA-based network structure modified with histones and myeloperoxidase (MPO). Furthermore, NaF led to the production of reactive oxygen species (ROS) in neutrophils. Western blot results showed that NaF significantly increased the phosphorylation of AMPK and p38. In addition, our results showed that NaF-induced NET formation could be inhibited by an AMPK or p38 inhibitor. In conclusion, our results showed that NaF induced NET formation in neutrophils through regulation of the AMPK/p38 signaling pathway.

## 1. Introduction

Fluoride is one of the essential microelements in the body. Long-term excessive intake of fluoride will cause systemic physiological and pathological changes [1]. Fluoride can not only damage bone organs but also accumulate in brain tissue through the blood-brain barrier, affecting the shape and function of brain nerve cells [2, 3]. In recent years, fluoride pollution is serious due to the development of industry. Meanwhile, increased fluorine in water will cause a certain toxic effect on aquatic animals [4]. Fish can absorb fluorine directly in water, which is an aquatic organism vulnerable to fluorine pollution in the water [5]. A previous study showed that sodium fluorine could affect the injury of brain tissues and behaviour of zebrafish in vivo [6]. Also, fluorine could cause thyroid endocrine disruption in male zebrafish [7]. Furthermore, fluorine could induce the injury of the gills of *Cyprinus carpio* [8]. In addition, fluorine has been reported to induce head kidney macrophage cell apoptosis in vitro [9].

Neutrophils are the most abundant white blood cells in the peripheral blood. It plays an important role in the innate immune system [10]. It is the first line in defending against the invasion of pathogens. In recent years, neutrophils have been found to have a new mechanism in resisting microbial invasion: the formation of neutrophil extracellular traps (NETs) [11]. NETs play an important role in pathogen infection, and its huge network structure can adhere, restrict, and kill pathogenic microorganisms [12]. However, the release of NETs is a double-edged sword, which not only destroys pathogenic microorganisms but also damages the organism [13]. Studies showed that NETs were involved in the pathological process of many diseases, such as lung injury, acute pancreatitis, inflammatory bowel disease, and arthritis [14–17]. Meanwhile, recent studies demonstrated that sodium arsenic, cadmium chloride, or nanosilver could induce the formation of NETs [18–20]. However, whether fluorine could induce the formation of NETs has not been reported. In the present study, the effects of fluorine on NET formation and its possible mechanism were investigated.

## 2. Materials and Methods

### 2.1. Reagents

Sodium fluoride (NaF) was purchased from Sigma-Aldrich (St. Louis, USA). A PicoGreen Quantitative kit was purchased from Invitrogen (CA, USA). A fish PMN isolation kit was provided from Tianjin Haoyang Biological Manufacture (Tianjin, China).

### 2.2. Neutrophil Isolation and Treatment

Healthy common carp (mean body weight, 500-550 g) were purchased from the Xinli Reservoir (Changchun, China). The blood was collected from the caudal vein of the individual fish after they were anesthetised with benzocaine. Neutrophils were isolated from the blood using the fish PMN isolation kit according to the manufacturer's instruction. Then, neutrophils were resuspended in RPMI 1640 medium (phenol-red free) and adjusted to 1 × 10^5^ cells/mL. The experiments were conducted according to the Chinese regulation for Experimental Animals and approved by the Institutional Animal Care and Use Committee of Jilin Agricultural University (#2019167).

### 2.3. Confocal Laser Scanning Fluorescence Microscopy Analysis

Neutrophils were seeded on poly-L-lysine pretreated coverslips at a density of 2 × 10^5^ cells in 96-well plates. Then, the cells were incubated with NaF (1 mM) or PBS at 37°C for 2 h. Then, the samples were fixed with 4% paraformaldehyde for 30 min and sealed with fetal bovine serum. After 3 h, NE (AB68672; Abcam) and MPO (Orb16003; Biorbyt) primary antibodies were incubated at 4°C overnight. The secondary antibody (goat anti-rabbit IgG-FITC conjugated, Bioworld Technology Inc.) was incubated at room temperature for 3 h, and SYTOX orange (5 *μ*M) was stained at room temperature for 15 min. Finally, images were taken using a scanning confocal microscope (Olympus FluoView FV1000).

### 2.4. Quantitation of NETs

The effects of NaF on NET formation were detected quantitatively by the PicoGreen kit according to the manufacturer's instruction. Neutrophils were added into 96-well plates and incubated with NaF (0.25, 0.5, and 1 mM) for 2 h. Zymosan (1 mg/mL) was used as the positive control. For the inhibitory experiment, the cells were pretreated with an AMPK inhibitor (10 *μ*M) or p38 inhibitor (SB202190, 10 *μ*M) for 20 min and then treated with NaF for 2 h.

### 2.5. ROS Measurement

Neutrophils were seeded into the 96-well plates and treated with DCFH-DA staining solution for 30 min. After washing with RPMI 1640 medium (phenol-red free), the cells were treated with NaF (0.25, 0.5, and 1 mM) for 2 h. Zymosan was used as the positive control group. After washing twice with phenol-red free 1640, the cells were detected by a fluorescence enzyme-labeled instrument Infinite M200 (TECAN, Austria). The excitation wavelength was 488 nm, and the emission wavelength was 535 nm.

### 2.6. SOD and CAT Activity Assay

The activities of SOD and CAT were detected using the detection kits according to the manufacturer's instructions (Nanjing Jiancheng Bioengineering Institute, Nanjing, China). Briefly, neutrophils were treated with NaF (0.25, 0.5, and 1 mM) for 2 h and harvested. Then, the cells were lysed by a lysis buffer and mixed with the regents according to operation tables. Finally, the samples were detected with an ultraviolet spectrophotometer (PERSEE, China).

### 2.7. Western Blot Analysis

Neutrophils were seeded into the 6-well plate and treated with NaF (0.25, 0.5, and 1 mM) for 2 h. Previous studies showed that these proteins are still phosphorylated when neutrophils were stimulated by toxins, such as Ochratoxin A, Fumonisin B1, and sodium arsenic [20–22]. Meanwhile, NET formation was detected 2 h after NaF stimulation. Therefore, we chose 2 h based on these previous published articles. Total protein was extracted using the Protein Extraction Reagent Kit (Beyotime Biotechnology, China). The protein concentration was measured by the BCA protein quantitative kit (Beyotime Biotechnology, China). The extracted samples were separated by 12% SDS-PAGE and transferred to the PVDF membrane. The membrane was sealed by 5% BSA at room temperature for 2 h. Then, the membrane was incubated with primary antibodies: p38 (Cell Signaling Technology, Cat# 8690), p-p38 (Cell Signaling Technology, Cat# 9215), p-AMPK (Beyotime, Cat#AA393), AMPK (Proteintech, Cat#10929-2-AP), and *β*-actin (Cell Signaling Technology, Cat# 8457) at 4°C overnight and the second antibody (1 : 10000, ImmunoWay, RS0001, RS0002) at room temperature for 2 h. The reactivity of the primary antibodies used in this study is against Cyprinus carpio. The ECL Plus Western Blotting detection system was used to detect the signal. Finally, the gray level was analyzed by ImageJ software.

### 2.8. Statistical Analysis

The data of this study were presented as mean ± SEM, and the differences between the groups were analyzed using SPSS 18.0 and detected by one-way ANOVA. Statistical significance was expressed as ^∗^*P* < 0.05 and ^∗∗^*P* < 0.01.

## 3. Results

### 3.1. Effects of NaF on the Formation of NETs

**Figure 1:**
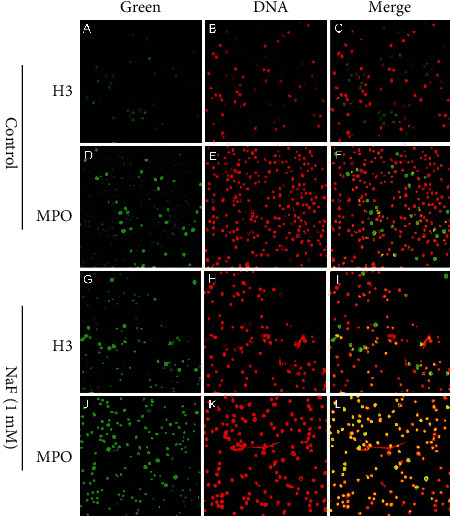
Visualization of DNA decorated with histones (H3) and neutrophil elastase (NE) in NaF induced NET structures. Neutrophils were stimulated with NaF (1 mM) or PBS (control group). And the formation of NETs was determined by fluorescence confocal microscopy. (b, e, h, k) show DNA. (a, g) show H3. (d, j) show MPO. (c) which is merged from (a, b) shows H3 in PBS-induced network structures. (f) which is merged from (d, e) shows MPO in PBS-induced network structures. (i) which is merged from (g, h) shows H3 in NaF- (1 mM) induced network structures. (l) which is merged from (j, k) shows MPO in NaF- (1 mM) induced network structures.

**Figure 2:**
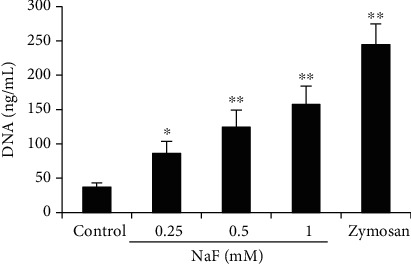
Quantitation of NETs. Cells were seeded into 96-well plates and incubated with NaF (0.25, 0.5, and 1 mM) for 2 h. Zymosan (1 mg/mL) was used as the positive control. NET release was quantified with Quant-iT™ PicoGreen dsDNA reagent. The data were presented as mean ± SEM, and statistical significance is expressed as ^∗^*P* < 0.05 and ^∗∗^*P* < 0.01.

The effects of NaF on NET formation were measured by immunofluorescence microscopy and a PicoGreen kit. Fluorescence colocalization confirmed that the network is a classic NET. The results showed that neutrophils released a network structure after the cells were treated with NaF, which is composed of DNA (red), NE (green), and MPO (green) (Figure 1). Meanwhile, NETs were quantified by PicoGreen, and the results showed that NaF could induce NET formation in a concentration-dependent manner (Figure 2).

### 3.2. Effects of NaF on ROS Production

**Figure 3:**
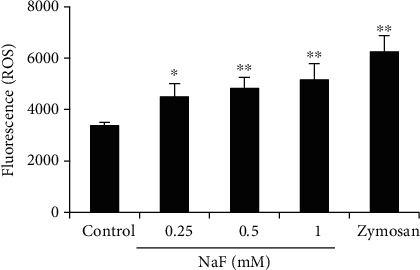
NaF induced ROS production. Neutrophils were seeded into the 96-well plates and treated with DCFH-DA staining solution for 30 min. After washing with RPMI 1640 medium (phenol-red free), the cells were treated with NaF (0.25, 0.5, and 1 mM) for 2 h. Zymosan was used as the positive control group. The data were presented as mean ± SEM and statistical significance is expressed as ^∗^*P* < 0.05 and ^∗∗^*P* < 0.01.

The effects of NaF on ROS production were detected in this study. As shown in Figure 2, NaF significantly increased the production of ROS, and this increase was in a concentration-dependent manner (Figure 3).

### 3.3. Effects of NaF on SOD and CAT Activities

**Figure 4:**
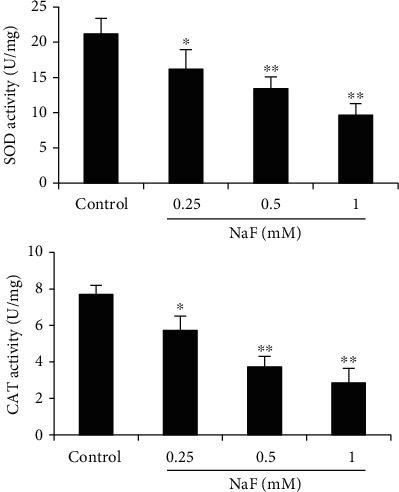
Effects of NaF on the activities of SOD and CAT. The data were presented as mean ± SEM, and statistical significance is expressed as ^∗^*P* < 0.05 and ^∗∗^*P* < 0.01.

SOD and CAT, important antioxidant enzymes, were detected in this study. As shown in Figure 4, NaF treatment significantly decreased the activities of SOD and CAT, and the decreases were in a concentration-dependent manner (Figure 4).

### 3.4. Effects of NaF on the Phosphorylation of AMPK and p38

**Figure 5:**
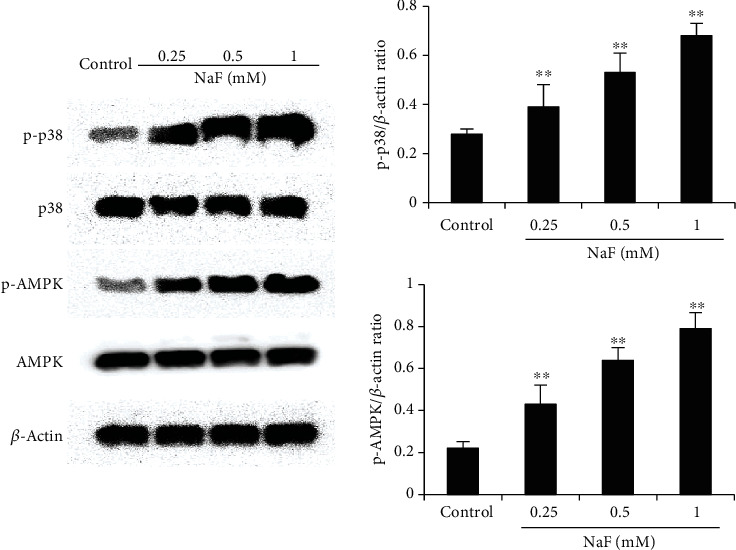
Effects of NaF on AMPK and p38 phosphorylation. The data were presented as mean ± SEM, and statistical significance is expressed as ^∗^*P* < 0.05, ^∗∗^*P* < 0.01.

AMPK and p38 have been reported to be involved in the regulation of NET formation. Therefore, we detected the effects of NaF on AMPK and p38 phosphorylation. As shown in Figure 5, the phosphorylation of AMPK and p38 increased significantly in the NaF-treated group than the control group.

### 3.5. NaF Induces NET Formation through AMPK/p38 Signaling Pathway

**Figure 6:**
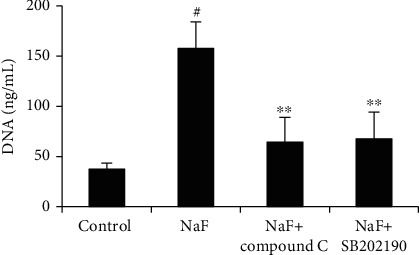
NaF induces NET formation through AMPK/p38 signaling pathway. Cells were incubated with indicated concentration of NaF (1 mM) for 120 min in the presence or absence of inhibitors. NET release was quantified with a Quant-iT™ PicoGreen dsDNA reagent. The data were presented as mean ± SEM, and statistical significance is expressed as ^#^*P* < 0.01 vs. the control group and ^∗∗^*P* < 0.01 vs. the NaF group.

To investigate the mechanism of NaF-induced NETs, the AMPK inhibitor (Compound C) or p38 inhibitor (SB202190) was used in this study. The results showed that NaF-induced NET formation was significantly attenuated by the AMPK inhibitor Compound C and p38 inhibitor SB202190 (Figure 6).

## 4. Discussion

NETs are a double-edged sword. Excessive production of NETs could lead to tissue injury. Experimental evidence suggests that NETs participate in the pathogenesis of autoimmune and inflammatory disorders. Recently, NETs have been reported to be involved in the pathological process of toxicant-induced injury. The production of NETs could aggravate poison-induced damage. Fluorine, an important environmental toxicant, is widely dispersed in the aquatic environment. However, whether fluorine could induce NET formation has not been reported. In the present study, we investigated the new effects of NaF on the NET formation of common carp neutrophils. Our results suggested that NaF-induced NET formation was through regulating the AMPK/p38 signaling pathway. These results can enrich the toxicological effect of fluoride.

In recent years, more and more reports showed that NETs could not only eliminate pathogenic microorganisms but also cause tissue damage and participate in the development of many diseases [13]. Furthermore, recent studies demonstrated that NETs were involved in the pathological process of toxicant-induced injury [20]. A previous study showed that di(2-ethylhexyl)phthalate (DEHP) could induce the formation of NETs in vitro [23]. Also, it has been reported that cadmium chloride could induce the formation of NETs, and inhibition NET formation had protective effects against cadmium chloride-induced lung injury in mice [19]. However, the effects of NaF on NET formation of common carp neutrophils have not been reported. In this study, our results showed that NaF could induce the formation of NETs in a concentration-dependent manner.

It has been reported that ROS and PAD4 are involved in the formation of NETs [24]. The formation of NETs is closely related to ROS, which is the product of NADPH oxidase activation [25]. Previous studies have shown that pretreatment with DPI, a NADPH oxidase inhibitor, significantly inhibited the formation of NETs [26]. ROS can activate the mitogen-activated protein kinase (MAPK) signal pathway and its downstream p38, thus promoting the production of NETs [27]. And a previous study showed that inhibition of p38 phosphorylation could prevent NET formation [22]. In this study, we found that NaF exposure significantly increased the production of ROS. Meanwhile, NaF could activate AMPK and p38 signaling pathways. To further clarify the formation mechanism of NETs, AMPK and p38 inhibitors were added to the cells. The results showed that NaF-induced NET formation was inhibited by these inhibitors, suggesting that NaF-induced NET formation was dependent on AMPK and p38 signaling pathways.

Taken together, the results of this study demonstrated that NaF significantly increased the release of NETs of neutrophils. The mechanism was through regulating the AMPK/p38 signaling pathway. The results of the present study could enrich the toxicity effects of fluorine on the immune system of the common carp.

## Data Availability

The data used to support the findings of this study are available from the corresponding author upon request.
